# Effect of simulation based workshop on changing faculty perception for simulation-based education

**DOI:** 10.12669/pjms.40.8.8598

**Published:** 2024-09

**Authors:** Rabia Aftab, Fouzia Kirmani, Tahir Ansari, Masood Ahmed

**Affiliations:** 1 Dr. Rabia Aftab, BDS. Department of Medical Education, Fazaia Ruth Pfau Medical College, Air University PAF Base Karachi, Pakistan; 2 Dr. Fouzia Kirmani, MBBS. Department of Medical Education, Fazaia Ruth Pfau Medical College, Air University PAF Base Karachi, Pakistan; 3 Dr. Tahir Ansari, MBBS, MCPS, FCPS, MRCP, FRCP, MCPS-HPE. Department of Medical Education, Fazaia Ruth Pfau Medical College, Air University PAF Base Karachi, Pakistan; 4 Dr. Masood Ahmed, MBBS, MPhil, PhD. Department of Medical Education, Fazaia Ruth Pfau Medical College, Air University PAF Base Karachi, Pakistan

**Keywords:** Simulation, Perception, Debriefing

## Abstract

**Objective:**

To compare the perceptions of participants before and after a one-day workshop on Simulation-based Education. The other objective was to determine the feedback of participants about the one day workshop on Simulation-based Education.

**Methods:**

In March 2023, a one-day workshop on Simulation-Based-Education (SBE) was conducted by the Department of Medical Education of Fazaia Ruth Pfau Medical Education in collaboration with the foreign guest faculty through zoom. This workshop was conducted with the participants (faculty members) of the Certificate program. The study adopted quasi experimental (pretest posttest) research design. For data collection we used a validated questionnaire which compromises of three parts. Data was analyzed using SPSS 23. This is a semi-structured questionnaire which consists of four parts. The first part entails the demographic data of the participants. The second structured part collects the perception of participants through 26 statements on 5 points Likert scale (strongly disagree = 1, disagree = 2, agree to some extent = 3, agree = 4, strongly agree = 5).

**Results:**

The mean difference in participant perceptions was significant (P<0.05)on 13 statements: Improves communication skills (pretest 3.05±1.050, posttest 4.20±1.056; *p=0.004*), enhance teamwork (pretest 3.30±0.979, posttest 4.30±0.923; *p=0.004)*, overcomes the challenge of uncooperative patients during real practice (pretest 3.80±0.696, posttest 4.30±0.470; *p=* 0.008), enact live patients (pretest 2.70±0.923, posttest 3.65±1.040; *p=*0.004), incopororation into medical education (pretest 3.20±0.894, posttest 4.40±0.503; *p=*0.000), provide safe, reliastic and conducive learning environment (pretest 2.85±0.875, postest4.00±0.795; *p=*0.000), provide easy learning (pretest 2.75±0.716, posttest 4.05±0.605 *p=*0.000), decrease ethical issues with more repeated practice (pretest 2.75±0.716, posttest 3.90±0.788; *p=*0.000), reduces the effort put in by a faculty in clinical teaching (pretest 2.80±0.696, posttest 3.45±0.999; *p=*0.039), supplement to clinical practice (pretest 2.75±0.444, posttest 4.55±0.510; *p=*0.000), evidence required for simulation activities (pretest 2.95±0.605, posttest 4.10±0.641; *p=0.000*), able to add simulation in clinical subject (pretest 3.15±1.089, posttest 3.80±0.834; *p=* 0.055), can instruct complex skills without simulation (pretest 2.55±0.887, posttest 3.40±0.883; *p=*0.018).

**Conclusions:**

The study signifiacnaty changed the faculty members’ perceptions of simulation-based education.These encouraging findings may influence their future practice in simulation-based education, allowing them to provide safe, high-quality health care in the workplace and, eventually, enhance patient outcomes.

## INTRODUCTION

Around the world, there have been considerable changes to medical education. Concern for the patient’s safety is one of the causes for the adjustments.^1^ Simulation-based healthcare education has grown significantly over the past few years. These changes signify a pivotal moment where simulation is no longer perceived as a novelty that requires defense by a small group of passionate advocates.^2^ Simulation is a method or technique that is used to create an experience without really going through the real event.^3^ This approach offers a chance to instruct, inform, educate, train, and coach healthcare workers using fictitious patients or aids.^4^ The discipline of simulation-based learning is expanding and provides medical professionals and students with a secure, regulated environment for learning. Since the invention of mannequins (also known as dummies) in the middle of the 17^th^ century, simulations have been a significant learning tool in medical and nursing education for almost 400 years. It was formerly only allowed for the practice of fundamental skills, but today it may be used to boost learner competency and patient safety, which can reduce medical errors and enhance team management abilities among trainers and students.^5^

Additionally, simulation has started to alter a lot of the ways that trainees and junior doctors are taught medicine and develop the necessary abilities. Using simulation technology, medical, nursing, and other healthcare personnel can refine their abilities repeatedly if necessary without endangering patients.^6^

Faculty create simulations to get the intended educational objectives. They design simulations as an immersive teaching strategy that imitates or replicates real-world situations, issues, processes, or competencies. Students participate in the situation, put their skills to use, think critically, and extrapolate lessons from it.The principles of simulation as a teaching strategy connect well with constructivist teaching and learning theory, which may be modified for social and physical learning experiences to fulfil the requirements of all students.^7^

SBE training needs to be incorporated into all health professions education levels.^8^ Understanding simulation-based education and applying it in an educational context requires faculty training. This is a new advancement in our country, and currently, our faculty is not adequately prepared to utilize it to its maximum potential. Faculty members need to be familiar with the benefits and drawbacks of simulation-based education and be equipped with various capacity-building activities to effectively integrate it into their teaching methods. Faculty members should have opportunities to learn and seek clarification for their questions.^9^ With adequate training of faculty, we will be able to effectively utilize simulation and yield excellent results.

For building capacity of our faculty members about simulation-based education, a one-day workshop on Simulation-Based-Education (SBE) was conducted by the Department of Medical Education of Fazaia Ruth Pfau Medical Education. The objectives of this research study were to:

1.Compare the perceptions of participants before and after a one-day workshop on Simulation-based Education
2.Determine the feedback of participants about the one day simulation based education workshop

## METHODS

A one-day workshop on Simulation-Based-Education (SBE), in March 2023, was conducted by the Department of Medical Education of Fazaia Ruth Pfau Medical Education in collaboration with the foreign guest faculty through zoom with the permission of the Principal and Director Medical Education, FPRMC. The duration of this workshop was five hours.Non probability convenience sampling was used and the medical education department decided to keep the participants 20-25 in number for participation in the one day programme.

### Inclusion & Exclusion Criteria:

Out of 25 registration, twenty participants were included as a part of this study.The participants who took part voluntarily were included in the study. The participants who missed any part of the questionnaire were excluded. For this research we adopted interventional study design (Pre test and Post test). The study was completed in 6 months after the data collected.

### Ethical Approval:

The permission from ethical review board of FRPMC (Ref: IRB/55; dated January 11, 2023) was aslo taken to utilize the data from the activities for our research study

### Data collection procedure:

The workshop consist of following activities and data was collected along with these:

### Activity 1 - Pre-test:

At the beginning of the workshop, the participants’ opinions about the SBE were recorded through a questionnaire that had been validated by Laerdal Global Health Nepal.^10^ This is a questionnaire which compromises of three parts. The participants’ demographic information is included in the first section. The second part collects the perception of participants through 26 statements on five points Likert scale. The likert scale was defined as strongly disagree = 1, disagree = 2, agree to some extent = 3, agree = 4, strongly agree = 5. The first and second part of this questionnaire was used in this activity. The data collected was termed as pre test data.

### Activity 2 - Online synchronous session:

After the pre-test, an interactive online synchronous session was conducted by the team of the Simulation Department of The Hospital for Sick Children. The team of multiple experts in the simulation field briefed the participants about the practice of simulation in the health setting through group discussion. All the information regarding the process of simulation and how to implement it in the health education settings were discussed with the participants. The experts offered participants the chance to ask questions, which were promptly addressed in real-time..

### Activity 3 – Jigsaw:

After the online synchronous session, the participants were provided with the reading material related to the process, implication, benefits, and shortcomings of the SBE in health sciences in the form of articles. The participants were then involved in a Jigsaw activity to do the reading of a particular topic within the group related to simulation and then share the information with the larger group to complete the entire picture of SBE.

### Activity 4 - Role-play:

Facilitators from the DME and four volunteers from the participants demonstrated the two clinical scenarios using a task trainer and full body manikin. High fidelity simulated environment was created through the help of using scenarios of an emergency setting (pediatric hypovolemic shock case and RTA case) along with role-play.

### Activity 5 – Debriefing:

After the demonstration of medical simulation, a debriefing session was conducted under the supervision of the facilitators from DME. Participants of role-play were asked to reflect on their performance and overall activity of simulation. Debriefing is the main component of the clinical simulation which facilitates the participants to critically think about their perceptions and assumptions regarding the act of simulation.^11^

### Activity 6 - Post-test:

After all the activities, the participant’s perception was again taken through the questionnaire used before in the pre-test. The data collected was termed as post test data. Part three of the questionnaire was used here to take the feedback regarding the SBE workshop. This part consist of 14 statements.

### Data Analysis Plan:

The data were analyzed by using SPSS 22. Descriptive analysis was done for frequency and means ± SD. When comparing responses from the pretest and posttest, paired t-tests were employed to compare means.

## RESULTS

### Demographic and educational information of participants:

Out of 25 participants, only 20 participants gave permission to fill out the questionnaire. Among these, 100% belonged to full-time faculty members at different institutes. There were more female participants (55%) than males (45%) as shown in Table-I.

**Table-I:** Demographic profile of the participants

Item	Frequency	Percentage %
***Employment status***		
Full time	20	100
Part time	00	00
***Age range (years)***		
25–35	09	45%
36–40	02	10%
41-45	3	15%
46–50	4	20%
>50	02	10%
***Sex gender***		
Male	09	45%
Female	11	55%
***Disciplines***		
Dental	02	10%
Pathology	04	20%
Gynecology	03	15%
Family Medicine	01	05%
Forensic Medicine	02	10%
ENT	01	05%
Biochemistry	02	10%
Other	05	25%
***Academic position***		
Lecturer	04	20%
Senior lecturer	04	20%
Assistant professor	08	40%
Professor	01	05%
Other	03	15%
***Teaching duration (years)***		
<5	13	65%
6-10	05	25%
11-25	01	05%
>25	01	05%

### Analysis of mean differences in perceptions of SBE:

Table-II displays the average scores of participants’ responses to statements about their perceptions of Simulation-Based Education (SBE) on a Likert scale during both the pretest and posttest are displayed in Table-II. Using paired t-tests with 95% confidence intervals and 16 degrees of freedom, the table presents the differences in mean scores before and after the workshop. A significance level of P < 0.05 was used, and it was found that 13 out of 26 statements showed statistically significant differences: Improves communication skills (pretest 3.05±1.050, posttest 4.20±1.056; *p=0.004*), enhance teamwork (pretest 3.30±0.979, posttest 4.30±0.923; *p=0.004)*, overcomes the challenge of uncooperative patients during real practice (pretest 3.80±0.696, posttest 4.30±0.470; *p=* 0.008), enact live patients (pretest 2.70±0.923, posttest 3.65±1.040; *p=*0.004), incopororation into medical education (pretest 3.20±0.894, posttest 4.40±0.503; *p=*0.000), provide safe, reliastic and conducive learning environment (pretest 2.85±0.875, postest4.00±0.795; *p=*0.000), provide easy learning (pretest 2.75±0.716, posttest 4.05±0.605 *p=*0.000), decrease ethical issues with more repeated practice (pretest 2.75±0.716, posttest 3.90±0.788; *p=*0.000), reduces the effort put in by a faculty in clinical teaching (pretest 2.80±0.696, posttest 3.45±0.999; *p=*0.039), supplement to clinical practice (pretest 2.75±0.444, posttest 4.55±0.510; *p=*0.000), evidence required for simulation activities (pretest 2.95±0.605, posttest 4.10±0.641; *p=0.000*), able to add simulation in clinical subject (pretest 3.15±1.089, posttest 3.80±0.834; *p=* 0.055), can instruct complex skills without simulation (pretest 2.55±0.887, posttest 3.40±0.883; *p=*0.018).

**Table-II:** Perception of participants regarding the SBE workshop

No.	Item	Pretest (mean ± SD)	Posttest (mean ± SD)	P-value
	Improves communication skills	3.05±1.050	4.20±1.056	0.004
	Enhance teamwork	3.30±0.979	4.30±0.923	0.004
	Facilitates the enhancement of clinical skills and the performance of practitioners.Top of Form	4.20±1.005	4.50±0.513	0.209
	Aids in comprehending and managing even the most uncommon cases.	4.00±0.795	4.25±0.639	0.287
	Overcomes the challenge of uncooperative patients during real life practice	3.80±0.696	4.30±0.470	0.008
	Reduce the stressful learning environment usually observe in wards	3.95±0.945	4.15±0.813	0.464
	Faciliatates in the assessment of students performance.	4.10±0.641	4.25±0.444	0.453
	Enhances patient safety	4.00±1.076	4.35±0.489	0.217
	Enact live patients in practical examinations	2.70±0.923	3.65±1.040	0.004
	Better opportunity to learn than beside teaching with live patients.	2.70±0.923	3.15±1.309	0.251
	Incopororation into medical education	3.20±0.894	4.40±0.503	0.000
	Enhances the confidence of students while dealing with live patients	3.35±0.671	3.90±0.912	0.061
	Provide safe, reliastic and conducive learning environment.	2.85±0.875	4.00±0.795	0.000
	Provide easy learning.	2.75±0.716	4.05±0.605	0.000
	Decrease ethical issues with more repeated practice.	2.75±0.716	3.90±0.788	0.000
	Reduces the effort put in by a faculty in clinical teaching	2.80±0.696	3.45±0.999	0.039
	Supplement to clinical practice, not a replacement	2.75±0.444	4.55±0.510	0.000
	Costly compared to employing a trained person	3.65±1.040	3.95±0.759	0.368
	Evidence required for simulation activities	2.95±0.605	4.10±0.641	0.000
	Interpersonal relationships are essential	3.00±1.170	3.50±1.395	0.268
	Able to develop rating scales for skills and attitude evaluation	3.35±0.875	4.05±0.605	0.330
	Able to add simulation in my clinical subject	3.15±1.089	3.80±0.834	0.055
	Able to develop checklists for skills and attitude evaluation	2.20±0.768	4.10±0.447	0.349
	I can instruct complex skills without simulation	2.55±0.887	3.40±0.883	0.018
	Immediate feedback is essential	3.85±0.988	3.90±0.788	0.867
	Materials and equipment should be prepared beforehand.	4.05±1.276	4.30±0.470	0.398

***Notes:*** Strongly disagree = 1; disagree = 2; agre to some extent = 3; agree = 4; strongly agree = 5. *P<0.05=Significant.

### Feedback on the SBE workshop from participants:

On a Likert scale, Table-III displays participant response on the SBE workshop: I found the session very interesting (4.30±0.571), the session on SBE was useful to me for future work (4.25±0.550), the scenario was relevant to my subject (4.00±0.649), what I learnt will be useful for teaching (4.00±0.973), the resource persons/facilitators were helpful and effective (4.20±0.523), the resource persons/facilitators answered all my questions (4.10±0.553) and the resource persons/facilitators were professional and courteous (4.15±0.745).

**Table-III:** Feedback for the SBE workshop from participants (n=20).

No.	Item	Mean± SD
	The workshop’s goal was accomplished.	3.85±0.875
	I had trouble coming up with scenarios.	2.40±0.598
	I lack confidence in my ability to create tools for evaluating abilities and attitudes.	2.65±0.875
	I found the session very interesting	4.30±0.571
	The session on SBE was useful to me for future work	4.25±0.550
	The scenario was relevant to my subject	4.00±0.649
	The session was difficult to understand	2.25±0.550
	The time available for this session was not sufficient	2.60±0.883
	I learned no new techniques/ideas	2.50±0.946
	What I learnt will be useful for teaching	4.00±0.973
	The resource persons/facilitators were helpful and effective	4.20±0.523
	The resource persons/facilitators answered all my questions	4.10±0.553
	The resource persons/facilitators were professional and courteous	4.15±0.745
	I did not practice the techniques well	2.30±0.571

Notes: Strongly disagree = 1; disagree = 2; agre to some extent = 3; agree = 4; strongly agree = 5.

## DISCUSSION

Globally, medical education has undergone rapid transformation in response to all current issues, countless factors have contributed to these shifts, such as changing societal demands and countless scientific and technological advancements brought on by the development of evidence-based medical knowledge.^12^

Our findings indicate that participants initially believes that simulation could not improve communication skills and team work. However, after the workshop, their perceptions changed, and they acknowledged that SBE could indeed enhance students’s communication and teamwork skills. The primary reason could be that the workshop encompassed various aspects related to communication and teamwork skills. This comprehensive approach likely facilitated better understanding among the participants. Sezgin & Bektas in their study stated that SBE proven to be an effective instructional method in enhancing communication and teamwork among health professional students.^13^

Simulation provides a safe and controlled environment where learners can practice without the risk of harming real patients. This reduces anxiety and allows learners to focus on building their skills, even if the patient is uncooperative. It enacts live patients, allowing learners to practice until they feel confident in managing live patients. The result also showed significant difference in perception regarding the use of simulation to overcome challenges faced by uncooperative patient during real life practice. Also, simulation facilitates in enacting live patients. Elshama SS et al. have identified that SBE has overcome the issues of patient safety and patient care during the training of students. He stated that during live patient interaction, problems arise in dealing with uncooperative patients and patient safety also compromises, which has been easily overcome by the use of simulated patients in SBE.^14^

The results of this study encourage the integration of SBE in curricula of medical education as SBE offers a very secure and practical learning environment and also makes learning easy for students. Ayaz O & Ismail FW also indicated that Health simulation should be part of Medical curricula as it provides a variety of clinical cases close to reality in a secure learning environment for the students. Students learn the required clinical competencies in an easy and accessible way.^15^

Simulation can indeed help decrease ethical issues related to repeated practice by providing a controlled and ethical learning environment. Simulation allows learners to practice without putting real patients at risk. This eliminates the ethical dilemma of potentially harming patients through repeated practice or inexperienced interventions. This was well perceived by the participants as shown by the results. A study by Alshehri et al. stated that faculy members considered that simulation is a useful method for decreasing ethical issues. The faculty also emphasized that use of simulation can minimize errors through rigorous training in safe environment.^16^,^17^

Simulation based education can facilitate students but direct dealing with actual patients will always be essential to make health professionals aware of the full complexity of clinical practice. Thus, SBE is not an attempt to replace actual patient training experiences but rather a complementing instructional modality. This finding is supported by evidence found in literature. 18,19 The findings also revealed some interesting insights. Participants lacked confidence in developing checklists or evaluation forms to assess their students. Additionally, they were not supportive of the idea that simulation facilitates the assessment of students’ performance. This highlights an area for further development in their training. Since simulation plays a significant role in assessment, faculty members must receive training to develop relevant assessment tools. Top of Form

The feedback results showed that participants were encouraged to use simulation activities in their teaching and learning. They were satisfied with the performance of facilitators and the resources available. They enjoyed the sessions as they found them to be interesting. However, the workshop’s allotted time was not long enough for the participants to construct useful scenarios and evaluation tools, let alone put their newfound knowledge into practice.This need to be taken under consideration while planning future activities for simulation based education.

### Limitations:

The study has small sample size. Also most participants were from one single institution.

## CONCLUSION

The study showed that the workshop significantly changes the faculty members’ perceptions of simulation-based education. The session piqued their curiosity. The training improved their awareness of how SBE increases communication skills, teamwork, and provides a replicable learning environment. These encouraging findings may influence their future practise in simulation-based education, allowing them to provide safe, high-quality health care in the workplace and, eventually, enhance patient outcomes.

### Authors Contribution:

**RA** conceived, designed and did statistical analysis & editing of manuscript, is responsible for integrity of research.

**FK** did data collection and manuscript writing

**TA and MA** did review and final approval of manuscript

All autors are responsible for the integrity of the study.
